# Tetrapropylammonium Hydroxide Treatment of Aged Dry Gel to Make Hierarchical TS-1 Zeolites for Catalysis

**DOI:** 10.1021/acs.cgd.2c01291

**Published:** 2023-02-09

**Authors:** Zhenyuan Yang, Yanan Guan, Lei Xu, Yangtao Zhou, Xiaolei Fan, Yilai Jiao

**Affiliations:** † Shenyang National Laboratory for Materials Science, Institute of Metal Research, Chinese Academy of Sciences, 72 Wenhua Road, Shenyang110016, China; ‡ School of Materials Science and Engineering, University of Science and Technology of China, 72 Wenhua Road, Shenyang110016, China; § Department of Chemical Engineering, School of Engineering, The University of Manchester, Oxford Road, ManchesterM13 9PL, United Kingdom; ∥ Nottingham Ningbo China Beacons of Excellence Research and Innovation Institute, University of Nottingham Ningbo China, 211 Xingguang Road, Ningbo315100, China

## Abstract

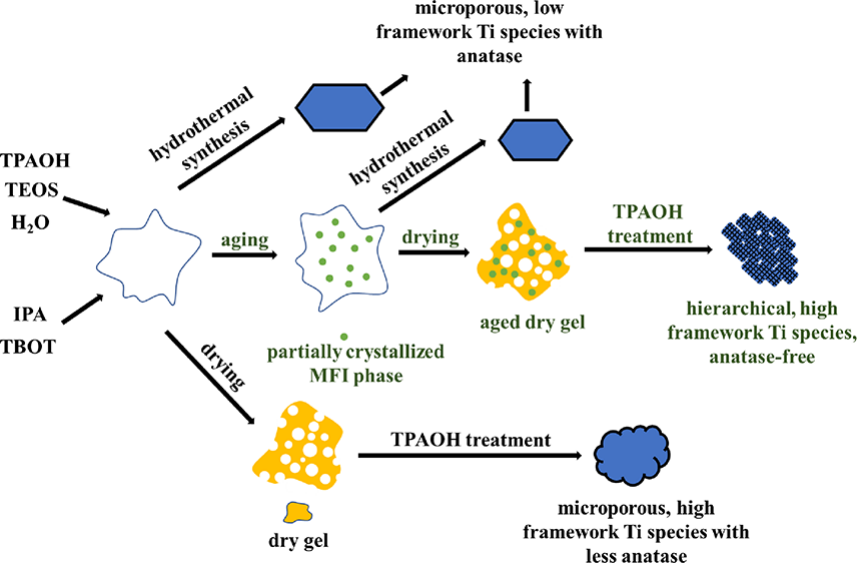

This work presents the development and systematic study of a method to prepare hierarchical titanium silicalite-1 (TS-1) zeolites with high tetra-coordinated framework Ti species content. The new method involves (i) the synthesis of the aged dry gel by treating the zeolite precursor at 90 °C for 24 h; and (ii) the synthesis of hierarchical TS-1 by treating the aged dry gel using tetrapropylammonium hydroxide (TPAOH) solution under hydrothermal conditions. Systematic studies were conducted to understand the effect of the synthesis conditions (including the TPAOH concentration, liquid-to-solid ratio, and treatment time) on the physiochemical properties of the resulting TS-1 zeolites, and the results showed that the condition of a TPAOH concentration of 0.1 M, liquid-to-solid ratio of 1.0, and treatment time of 9 h was ideal to enable the synthesis of hierarchical TS-1 with a Si/Ti ratio of 44. Importantly, the aged dry gel was beneficial to the quick crystallization of zeolite and assembly of nanosized TS-1 crystals with a hierarchical structure (*S*_ext_ = 315 m^2^ g^–1^ and *V*_meso_ = 0.70 cm^3^ g^–1^, respectively) and high framework Ti Species content, making the accessible active sites ready for promoting oxidation catalysis.

## Abstract 2

A new method based on aged dry gel and tetrapropylammonium hydroxide hydrothermal treatment was developed to synthesize nanosized TS-1 zeolite crystals with a hierarchical structure and high tetra-coordinated framework Ti species, which showed excellent catalytic performance in oxidation catalysis.

## 1 Introduction

Titanium silicalite-1 (TS-1) is an excellent oxidation catalyst for many important industrial relevant reactions such as alkene epoxidation,^1,2^ benzene hydroxylation,^3,4^ oxidative desulfurization,^5,6^ and synthesis of cyclohexanone oxime.^7,8^ The catalytic properties of TS-1 are mainly affected by two aspects, that is, (i) the intrinsic microporous framework of TS-1 (i.e., 0.54 × 0.56 nm, which hinders bulky molecules transport and accessibility to internal active sites), and (ii) the availability of catalytically active sites in TS-1. Regarding the latter, the framework Ti species are the active sites for catalysis, and conversely, the presence of anatase TiO_2_ phase (which is inactive for catalysis) is undesirable. Incorporation of more framework Ti species in TS-1 is challenging in conventional hydrothermal synthesis because anatase TiO_2_ is prone to formed due to the difference in the hydrolysis kinetics of tetraethyl orthosilicate (TEOS) and tetrabutyl orthotitanate (TBOT).^9^ In oxidation reactions using hydrogen peroxide (H_2_O_2_) over TS-1, the presence of TiO_2_ in TS-1 can result in the ineffective deposition of H_2_O_2_, thus reducing the catalytic efficiency.^10^ Accordingly, new methods for preparing TS-1 with excellent mass transfer properties and high framework Ti content are needed to improve its industrial applications.

Previously, considerable efforts have been devoted to improving mass transfer properties of microporous zeolites by introducing secondary auxilitigaoary meso-/macroporous pore networks into the intrinsic microporous zeolitic frameworks.^11,12^ If the two levels of pore systems are interconnected with certain hierarchy, the resulting zeolitic materials are called hierarchical zeolites. For making hierarchical TS-1, the use of mesoporogens can also hinder the insertion of Ti species into the framework during synthesis.^13^ Hence, alkaline postsynthetic treatments in the presence of tetrapropylammonium hydroxide (TPAOH) were developed for preparing hollow TS-1 with large pores.^14,15^ During the treatment, Si and Ti species in the parent TS-1 crystal dissolved and recrystallized with the assistance of TPAOH to form hollow structures which may benefit mass transfer. Importantly, such a dissolution and recrystallization process also enabled the reinsertion of the extraframework Ti and anatase TiO_2_ species back into the newly formed framework, hence improving the catalytic activity.^16,17^ However, this method is prone to produce closed meso-/macropores in TS-1 crystals, as evidenced by the poor activity in epoxidation of cyclohexene in comparison with that achieved by hollow TS-1.^14^ Recently, we have systematically studied the effects of the TPAOH concentration and the Si/Ti ratio of the parent TS-1 on the framework Ti content, crystal shape, secondary pore structure, and pore volume of the resulting materials from the post-treatment method. The findings suggest that the treatment using 0.5 M TPAOH and the parent TS-1 with a Si/Ti ratio of 46 can facilitate the conversion of extraframework Ti into the framework ones and create TS-1 with interconnected open meso-/macropore in TS-1. Unfortunately, the method is not suitable for treating the parent TS-1 with lower Si/Ti ratios of <46 since the framework Ti in the parent can hinder the dissolution/desiliconization process under alkaline conditions.^18^ The same issue was also encountered during the alkaline post-treatment of ZSM-5 zeolites.^19^ To solve this issue, we proposed a strategy to synthesize the parent ZSM-5 with low Si/Al ratios containing unprotected extraframework Al through rapid aging of the zeolite sol gel during the synthesis of the parent. The presence of the extraframework Al in the parent zeolite was found to be beneficial to encourage the dissolution and recrystallization processes during the post-treatment using TPAOH, as well as its conversion to framework Al in the resulting materials with the mesoporous hollow structure and low Si/Al ratios.^20^

Since TS-1 and ZSM-5 have the same MFI-type topology, the strategy may be translated to make hierarchical TS-1 with high framework Ti species content to improve catalytic activity. Zeolite precursor aging at low temperatures can give the precursor certain properties of zeolite, such as being microporous, which can be used as a seed to promote the fast nucleation and crystallization of zeolite.^21^ Serrano et al. prepared an aged zeolite precursor which was used to prepare TS-1 and ZSM-5 nanocrystalline aggregates by silanization.^22,23^ Recently, Yu et al. employed the aged precursor as seeds for the synthesis of single-crystal hierarchical ZSM-5 zeolites with hexagonal mesopores faceted by microporous domain. The aged precursor played a key role in the formation of faceted mesopores via intraparticle ripening process.^21^ Hence the aged precursors show promise to synthesize novel hierarchical zeolites.

Herein, this work presents the development of a novel yet simple method to prepare hierarchical TS-1 with high framework Ti content. To enable this, the aged dry gel was first prepared with abundant extraframework Ti species by aging the precursor at 90 °C for 24 h, which was subsequently used by TPAOH treatment to synthesize hierarchical TS-1 crystals with high framework Ti content. During the development, systematic investigation was conducted to vary the TPAOH concentration and liquid-to-solid (dry gel) ratio to control the recrystallization of the dry gel in the microzone to promote the transformation of extraframework Ti species into framework ones in the formed hierarchical TS-1 crystals. Relevant physiochemical properties of the materials at different stages of synthesis were carefully characterized, and the resulting hierarchical TS-1 zeolites were assessed by phenol hydroxylation and dibenzothiophene (DBT) desulfurization reactions to establish the synthesis-property-performance correlation. Findings of the work show that the developed strategy is simple and cost-effective to make framework Ti-rich hierarchical TS-1 crystals with excellent catalytic properties.

## 2 Experimental Section

### 2.1 Chemicals

For zeolite synthesis, the following chemicals are used as received: tetraethylorthosilicate (TEOS) (AR, Sinopharm), tetrabutyl orthotitanate (TBOT) (99%, Aladdin), tetrapropylammonium hydroxide (TPAOH) (25 wt %, Sinopharm), and isopropanol (IPA) (99%, Sinopharm). The prepared zeolites were assessed using phenol hydroxylation and dibenzothiophene (DBT) desulfurization, which used H_2_O_2_ (30 wt %, Sinopharm), phenol (Analytical reagent, AR, Alfa), DBT (99%, Macklin), *tert*-butyl hydroperoxide (TBHP) (65 wt %, Sinopharm), *n*-octane (CP, 95%, Macklin), and octadecane (99%, Adamas).

### 2.2 Synthesis of Materials

#### 2.2.1 Synthesis of the Aged Precursor Dry Gel

The zeolite precursor with the molar composition of 1.0SiO_2_: 0.025TiO_2_: 0.33TPAOH: 0.83IPA: 30H_2_O was prepared first. In detail, 80.79 g of deionized water was mixed with 65.08 g of TPAOH (25 wt %), and then 50 g of TEOS was added to the mixture under stirring (at 500 rpm) for 40 min to give a clear solution. Then, 2.04 g of TBOT was dissolved in 12 g of IPA, and the resulting solution was dripped to the solution above. The mixture was stirred (at 500 rpm) for 30 min to give a clear solution which was aged under reflux at 90 °C for 24 h. After aging, the resulting product was dried at 100 °C for 24 h until the mass of the gel remained unchanged. Subsequently, the dry gel was crushed to small particles with a size of 150 to 200 mesh, denoted as TPA@ST(90 °C). And the TPA@ST(90 °C) calcinated at 550 °C for 6 h was denoted as ST(90 °C).

#### 2.2.2 TPAOH Treatment of the Aged Dry Gel

To prepare hierarchical TS-1 zeolites, 3 g of TPA@ST(90 °C) was mixed with aqueous TPAOH solution and transferred to a PTFE-lined autoclave (100 mL) for further crystallization at 170 °C for 2–24 h. After the synthesis, the reaction mixture was centrifuged to separate the solid product (from the liquid), which was then washed using deionized water 3 times and dried at 100 °C in an oven overnight. After drying, the solid sample was calcined at 550 °C for 6 h to give the hierarchical TS-1 zeolites, denoted as HTS-*x*-*y*-*z*H, in which *x*, *y*, and *z* represent the TPAOH concentration, mass ratio of TPAOH solution to dry gel (or the liquid-to-solid ratio), and crystallization time (*x* = 0.1, 0.3, 0.5, 0.7 M; *y* = 0.5, 1.0, 1.7, 5.0; *z* = 2–24 h), respectively.

#### 2.2.3 Synthesis of Reference Samples

(1) TPAOH treatment of the unaged dry gel. To know the effect of the aging process on the properties of the obtained TS-1, a reference dry gel was synthesized using the procedure in Section 2.2.1, but omitting the aging step, denoted as TPA@ST. TPA@ST was treated with 0.1 M TPAOH solution at a liquid-to-solid ratio of 1.0 and a reaction temperature of 170 °C for 9 h. The resulting sample was defined as CTS-0.1-1.0-9H.
(2) Conventional hydrothermal treatment of the aged and unaged zeolite precursor. In addition, the aged and unaged zeolite precursor (without drying) were used directly in hydrothermal synthesis at 170 °C for 72 h to give relevant TS-1 zeolites, that is, CTS-1 obtained from the synthesis using the unaged precursor and NTS-1 obtained from the synthesis using the aged precursor, respectively. The same workup procedure was applied during the synthesis of CTS-1 and NTS-1.

### 2.3 Characterization of Materials

Powder X-ray diffraction (XRD) patterns of the materials were collected using a PANalytical X’Pert Multipurpose X-ray diffractometer with a scan step size of 0.02° per step at the rate of 147.4 s step^–1^, and the relative crystallinity (RC) of the samples was obtained by comparing the intensity of relevant characteristic peaks with the highest intensity of the materials under investigation. Nitrogen (N_2_) physisorption isotherms were collected at −196.15 °C using a Micromeritics 3Flex gas analyzer to investigate the porous properties of the materials. Prior to the analysis, all samples were degassed at 350 °C for 8 h. The Si/Ti ratio of the materials was determined by ICP-AES (Agilent 5800). Prior to the ICP-AES analysis, 10 mg of the TS-1 zeolite sample was dissolved in a 1 mL solution of 3:1:1 HCl: HNO_3_: HF at room temperature for 30 min, which was subsequently diluted to 100 mL with deionized water. To study surface morphology of the materials, SEM analysis was conducted using a Apreo at an accelerating voltage of 20 kV and working distance of 10 mm equipped with an energy dispersive X-ray spectrometer (EDX). Selected samples were also examined by HRTEM (FEI Tecnai F20 G2) at 200 kV. FT-IR spectra of the materials were recorded on a Nicolet 460 spectrometer at room temperature in KBr pellets over the range of 400–1600 cm^–1^ under the atmospheric conditions. The UV–vis diffuse reflectance spectra of the samples were collected on a JASCO V-770 UV–vis spectrometer over a range of 200 to 600 nm, and baseline correction was carried out using powder BaSO_4_. TGA of the materials was conducted by NETZSCH-STA449C TG/DTA Instruments under air at 100 mL min^–1^ with a ramp rate of 10 °C min^–1^. The magic-angle spinning (MAS) solid state nuclear magnetic resonance (NMR) study was carried out on a Bruker 400 M NMR spectrometer under ambient conditions.

### 2.4 Catalysis

The catalytic activity of the selected TS-1 samples (including HTS-*x*-*y*-*z*, CTS-0.1-1.0-9H, NTS-1, and CTS-1) were assessed by phenol hydroxylation and DBT desulfurization, which were carried out in a 50 mL two-necked round-bottom flask equipped with a reflux condenser. For phenol hydroxylation, deionized water (10 mL), phenol (2 g), H_2_O_2_ (720 μL; the molar ratio of phenol and H_2_O_2_ is 3:1), and TS-1 (100 mg) were charged into the flask, and the reaction was conducted under stirring (500 rpm) at 80 °C for 60 min. The liquid products were withdrawn during the reaction and extracted with ethyl acetate and analyzed by gas chromatograph (GC-Agilent 8890) equipped with the 30 m capillary column (HP-5) and FID detector. For DBT desulfurization, *n*-octane (10 mL, containing 3000 ppm DBT), TBHP (75 μL, the molar ratio of TBHP and DBT is 3:1, as the oxidizing agent), octadecane (as the internal standard in gas chromatography, GC, analysis), and TS-1 (50 mg) were mixed in the flask for the reaction under stirring (500 rpm) at 60 °C for 90 min, and the reaction mixture was analyzed using the GC above. The details of the GC methods were described elsewhere.^24,25^

Turnover number (TON) values of the catalytic systems were calculated using eq 1.^26^
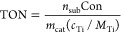
1where *n*_sub_ (mol) is the molar number of the substrate, Con (%) is the substrate conversion, *m*_cat_ (g) is the mass of the catalyst, *c*_Ti_ (wt %) is the titanium content in the catalyst (by ICP), and *M*_Ti_ is the atomic weight of Ti at 47.867 g mol^–1^.

## 3 Results and Discussion

### 3.1 Physiochemical Properties of Relevant TS-1 Zeolites

**Figure 1:**
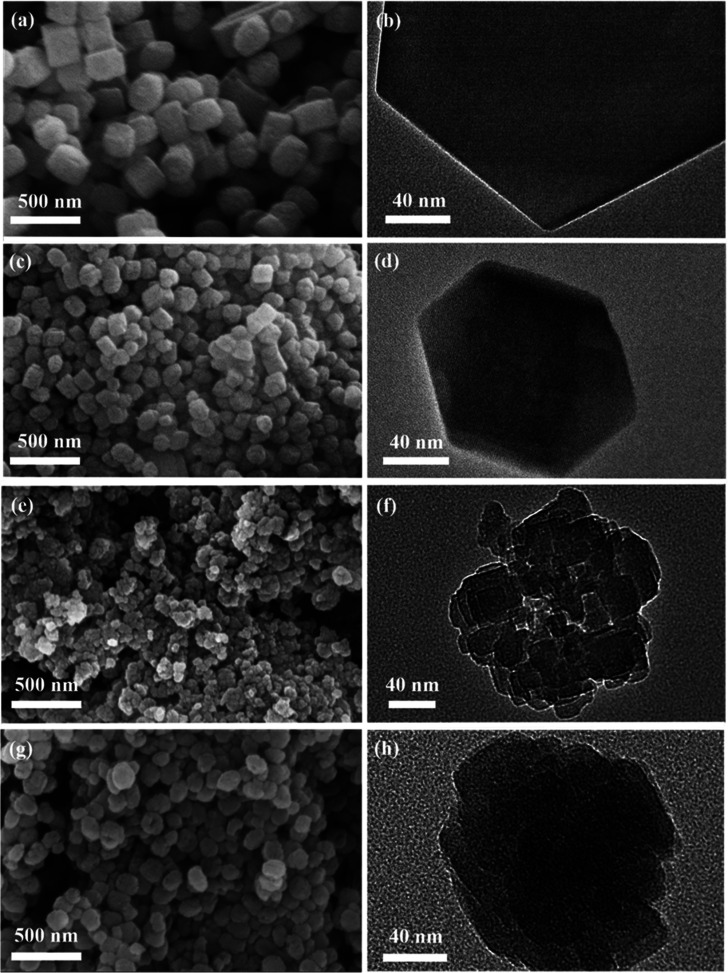
SEM and TEM imagines of (a, b) CTS-1, (c, d) NTS-1, (e, f) HTS-0.1-1.0-9H, and (g, h) CTS-0.1-1.0-9H.

Morphologies of the TS-1 samples synthesized by different methods were first studied by SEM and TEM (Figure 1). For the TS-1 samples prepared by the conventional hydrothermal method, i.e., CTS-1 and NTS-1, their crystals exhibit a typical MFI type zeolite hexagonal morphology without mesopores, as shown in Figure 1a–d. Comparatively, NTS-1 (based on the aged gel) has smaller crystal sizes, i.e., ∼130 nm for NTS-1 vs ∼270 nm for CTS-1. The difference in sizes could be attributed to the aged zeolite precursor (aged at 90 °C for 24 h) which acted as the seed to accelerate the rate of nucleation and crystallization.^3,27^ After the TPAOH treatment, the sample synthesized using the aged dry gel, viz. HTS-0.1-1.0-9H, shows the hierarchical structure consisting of the assemblies of small crystals of 20–30 nm (Figure 1e,f), suggesting the presence of intracrystalline mesopores. Conversely, after the treatment of the unaged gel, the resulting CTS-0.1-1.0-9H seems to not have mesopores in its crystals (Figure 1h, showing denser and more uniform TEM morphology compared to that of HTS-0.1-1.0-9H). Such findings suggest that the aging step during the dry gel preparation is critical to the formation of the hierarchical structure during the TPAOH treatment.

**Figure 2:**
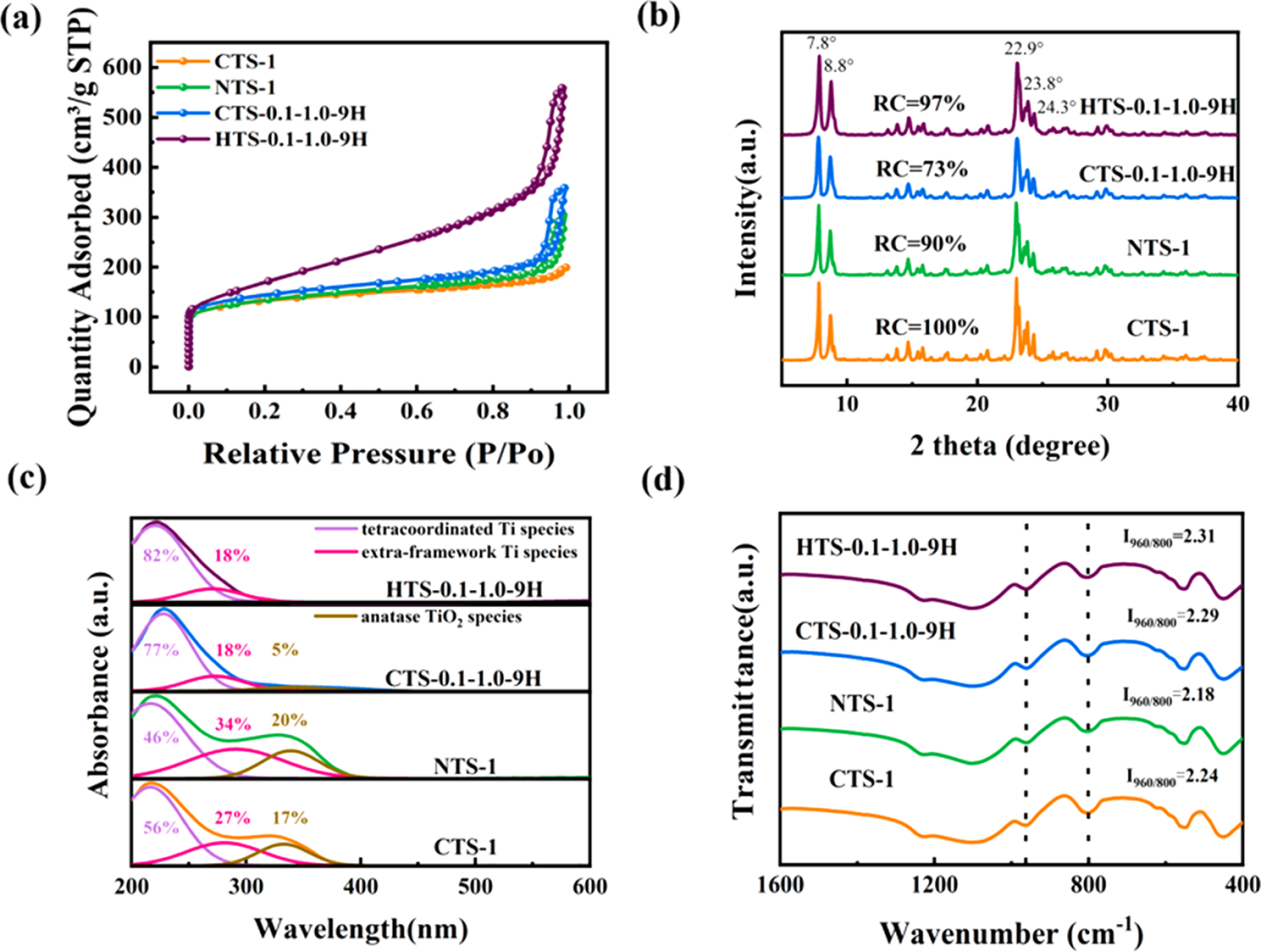
(a) N_2_ adsorption–desorption isotherms, (b) XRD patterns, (c) UV–vis spectra, and (d) FT-IR spectra of CTS-1, NTS-1, CTS-0.1-1.0-9H, and HTS-0.1-1.0-9H.

N_2_ physisorption analysis was performed to know the pore structures of CTS-1, NTS-1, CTS-0.1-1.0-9H, and HTS-0.1-1.0-9H. Relevant adsorption–desorption isotherms and pore size distributions (PSDs) of the TS-1 samples are shown in Figure 2a and Figure S1, respectively. In detail, CTS-1 shows the type-I isotherm, being a microporous material. Comparatively, NTS-1, CST-1, and HTS-0.1-1.0-9H all show the type-IV isotherm with apparent hysteresis loops in the high relative pressure range of 0.8 < *P*/*P*_0_ < 0.99), arising from the mesopores and/or macropores formed by packing of nanosized zeolite crystals.^5^ However, HTS-0.1-1.0-9H displays a pronounced uptake of N_2_ molecules at 0.2 < *P*/*P*_0_ < 0.8, indicating the presence of small mesopores, which can be due to the intracrystalline mesopores in the assembly of secondary units nanocrystalline with diameters of 20–30 nm. As shown in Figure S1, the PSD of HTS-0.1-1.0-9H shows a mesopore distribution at 1–10 nm, which is consistent with the findings by TEM. The textural properties of the TS-1 samples under investigation are shown in Table 1. Compared to CTS-1, NTS-1 and CTS-0.1-1.0-9H, HTS-0.1-1.0-9H exhibits the largest external surface area (*S*_ext_) of 315 m^2^ g^–1^ and the largest mesopore volume (*V*_meso_) of 0.70 cm^3^ g^–1^, which indicates that the aging process was essential for generating intracrystalline mesopores. The porous properties are also compared with relevant reported samples in the literature (Table S3), and HTS-0.1-1.0-9H possesses the largest external surface area and the largest mesopore volume.

**Table 1:** Textural Properties of the TS-1 Zeolites under Investigation

sample	Si/Ti^a^ [−]	*S*_BET_^b^[m^2^ g^–1^]	*S*_micro_^c^[m^2^ g^–1^]	*S*_ext_^c^[m^2^ g^–1^]	*V*_micro_^c^[cm^3^ g^–1^]	*V*_meso_^d^[cm^3^ g^–1^]
HTS-0.1-1.0-9H	44	584	269	315	0.17	0.70
CTS-0.1-1.0-9H	41	522	337	185	0.14	0.42
CTS-1	34	483	409	74	0.18	0.12
NTS-1	33	485	366	119	0.16	0.31
HTS-0.1-1.7-24H	41	542	340	202	0.17	0.47
HTS-0.3-1.7-24H	42	554	344	209	0.16	0.50
HTS-0.5-1.7-24H	47	553	324	229	0.15	0.48
HTS-0.7-1.7-24H	52	557	301	256	0.15	0.49
HTS-0-1.7-24H	41	546	349	197	0.14	0.47
HTS-0.1-0.5-24H	42	566	236	330	0.15	0.68
HTS-0.1-1.0-24H	41	576	230	346	0.17	0.66
HTS-0.1-5.0-24H	45	538	343	195	0.16	0.43
ST(90 °C)	43	765	507	258	0.26	0.3
HTS-0.1-1.0-2H	51	803	313	489	0.21	0.56
HTS-0.1-1.0-3H	47	597	319	278	0.14	0.46

a By ICP.

b By the Brunauer–Emmett–Teller (BET) method.

c By the *t*-plot method.

d By the Barrett–Joyner–Halenda (BJH) method (using the adsorption isotherm).

Powder XRD patterns of the TS-1 zeolites discussed above (Figure 2b) show the typical diffraction peaks of the MFI structure.^28^ Comparatively, CTS-1 shows the highest peak intensity, which was used as the reference (with RC = 100%) for determining the RC values of other samples, and CTS-0.1-1.0-9H shows a low RC value of 73%. Comparatively, the other two samples prepared using the aged dry gel, regardless the synthesis method, showed higher RC values, viz. 90% for NTS-1 and 97% for HTS-0.1-1.0-9H, showing the important role played by the aging process in zeolite crystallization during the TPAOH treatment of the gel. UV–vis spectroscopy was conducted to investigate the coordinate states of Ti species in the samples. As shown in Figure 2c, the absorption band at around 220 nm is attributed to the tetracoordinated Ti species, while the absorption band at 260–280 and 330 nm is assigned to the extraframework Ti species and anatase TiO_2_ species, respectively.^29^ According to the UV–vis spectra of the two TS-1 samples prepared by the conventional synthesis, a significant presence of extraframework Ti and anatase TiO_2_ species was identified, that is, about 54% for NTS-1 and 44% for CTS-1. Comparatively, framework Ti species are the dominant ones in the two samples synthesized using the TPAOH treatment, representing about 82% and 77%, respectively, for HTS-0.1-1.0-9H and CTS-0.1-1.0-9H. However, anatase TiO_2_ (about 5%) was also found in CTS-0.1-1.0-9H. The results from UV–vis spectroscopy suggest that (i) TPAOH treatment was very effective to enable the formation of framework Ti species, and (ii) the aging step during gel synthesis is necessary to prevent the formation of the anatase TiO_2_ phase during the TPAOH treatments. The Ti coordinate states in the four samples were also studied by FT-IR (Figure 2d). The absorption peak at 960 cm^–1^ is due to the vibration of the Si–O–Ti bond or the vibration of Si–O–Si closely connected with Ti–O–Si, while the absorption peak at 800 cm^–1^ is attributed to the characteristic peak of the MFI topology.^30,31^ The relative intensity ratio of the two peaks at 960 and 800 cm^–1^ (*I*_960/800_) can be used to assess the content of framework Ti species in the zeolitic frameworks; i.e., a relatively large value of *I*_960/800_ suggests more framework Ti species in the framework.^32,33^ As shown in Figure 2d, the *I*_960/800_ values of these samples rank as HTS-0.1-1.0-9H > CTS-0.1-1.0-9H > CTS-1 > NTS-1, which confirms the findings by UV–vis. In addition, ^29^Si NMR spectroscopy in Figure S2 also proved the high framework content Ti species in HTS-0.1-1.0-9H.

### 3.2 Systematic Study of the Synthesis of Hierarchical TS-1

**Figure 3:**
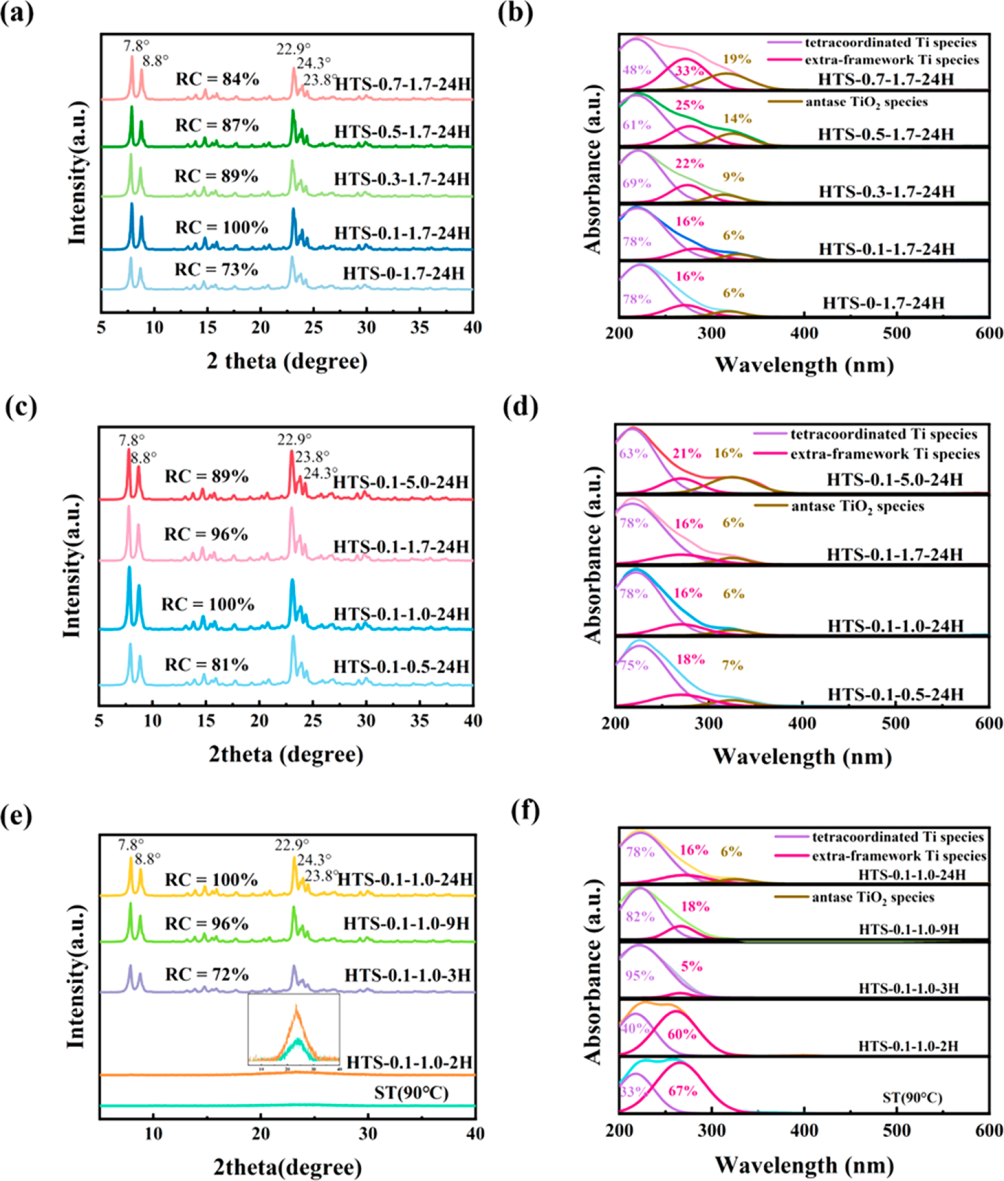
(a) XRD patterns and (b) UV–vis spectra of HTS-*x*-1.7-24H and HTS-0-1.7-24H; (c) XRD patterns and (d) UV–vis spectra of HTS-0.1-*y*-24H; (e) XRD patterns and (f) UV–vis spectra of HTS-0.1-1.0-*z*H.

To understand the effect of process parameters of the TPAOH treatment (including the TPAOH concentration and synthesis time) on the texture properties and coordinate states of Ti species, systematic investigation was performed accordingly. With a fixed treatment time of 24 h and a TPAOH solution to dry gel mass (liquid-to-solid) ratio of 1.7, the TPAOH concentration was varied from 0.1 to 0.7 M, and a control sample of HTS-0-1.7-24H was also prepared without TPAOH (i.e., the dry gel was treated using water only). As shown in Figure 3a, XRD analysis confirms that all the samples obtained have the typical MFI crystal structure. Compared to the control of HTS-0-1.7-24H, low-concentration TPAOH (0.1 M) can significantly increase the RC of the resulting sample, i.e., HTS-0.1-1.7-24H. However, with a further increase in TPAOH concentration, the RC of the resulting samples decreases. The UV–vis spectra of HTS-0-1.7-24H and HTS-0.1-1.7-24H are similar, and both exhibit higher tetracoordinated Ti species content and lower extraframework Ti and anatase TiO_2_ species contents in comparison with the other samples. An increase of TPAOH concentration led to the increase in the content of extraframework Ti and anatase TiO_2_ species, as shown in Figure 3b. The textural properties of the HTS-*x*-1.7-24H materials (Figure S3 and Table 1) show that the samples obtained by TPAOH treatment have a relatively larger external surface area compared to HTS-0-1.7-24H. Morphologies (by SEM, Figure S4) of the materials above reveal that the shape of the HTS-*x*-1.7-24H materials is more regular than that of HTS-0-1.7-24H, and an increase in TPAOH concentration increased the crystal size and made the crystal shape more regular. Based on the characterization results above, one can see that the treatment using 0.1 M TPAOH obtained TS-1 zeolite with high RC, high framework Ti species, and large mesopore volume and external surface being the most appropriate TPAOH concentration. The treatment with 0.1 M TPAOH allowed the fast nucleation and crystallization of TS-1, which was beneficial to the crystallinity and the insertion of Ti species into the framework. However, when relatively concentrated TPAOH (>0.1 M) was used, dissolution could be promoted, which could inhibit the insertion of the dissolved Ti species back into the framework, reducing the framework Ti content and RC. This hypothesis can be confirmed by comparing the Si/Ti ratios of the materials, compared to HTS-0.1-1.7-24H (Si/Ti = 41); an increase in TPAOH concentration caused the decrease in Si/Ti ratios of the resulting TS-1 samples, confirming that high TPAOH concentration was adverse for the reinsertion of Ti species into the framework during the treatment of dry gel.

With a fixed TPAOH concentration of 0.1 M and treatment time of 24 h, the effect of liquid-to-solid ratio of the properties of the resulting materials was studied. XRD analysis of the obtained HTS-0.1-*y*-24H materials (Figure 3c) shows that a moderate liquid–solid ratio of 1.0 promoted the comparatively best crystallinity. UV–vis spectra of HTS-0.1-*y*-24H (Figure 3d) show that the high liquid-to-solid ratio of 5.0 encouraged the formation of extraframework Ti and anatase TiO_2_ species. Also, N_2_ physisorption analysis (Figure S5 and Table 1) shows that HTS-0.1-5.0-24H has the lowest *S*_ext_ of 195 m^2^ g^–1^ and *V*_meso_ of 0.43 cm^3^ g^–1^. SEM analysis shows that the crystal morphologies of the samples treated under the condition of low liquid-to-solid ratios (<5.0) are relatively consistent, showing hierarchical structures assembled by small TS-1 crystals (150 nm). Regarding HTS-0.1-5.0-24H, its crystal size was much larger at ∼270 nm (Figure S6). The results above regarding the effect of the liquid-to-solid ratio on the properties of HTS-0.1-*y*-24H indicate that the conditions with high liquid-to-solid ratios provide excessive water and OH^–^ to promote the complete dissolution of the dry gel during the post-treatment, making the reaction mechanism close to that of the liquid-phase mechanism,^28^ and hence leading to the formation of large TS-1 crystals with anatase TiO_2_. It was also found that the liquid-to-solid ratio has less impact on the Si/Ti ratio of HTS-0.1-*y*-24H.

By maintaining the identified optimum TPAOH concentration (0.1 M) and liquid-to-solid ration (1.0) constant, the treatment time was varied to understand its effect on the properties of the resulting HTS-0.1-1.0-*z*H materials. TGA of TPA@ST(90 °C) (Figure S7, Table S1) shows that it consists of 74.4 wt % dry gel and 29.64 wt % TPAOH and 5.96 wt % water. XRD patterns (Figure 3e) of the pristine dry gel ST(90 °C) show the very weak characteristic diffraction peaks around 23°, which shows the amorphous feature of the aged dry gel with partial MFI structure.^21,35,36^ After a 2-h TPAOH treatment, the intensity of the characteristic peaks was enhanced in HTS-0.1-1.0-2H, indicating the increase in the crystallinity in the solid phase.^21^ By increasing the treatment time to 3 h, crystallization to the MFI structure was achieved with HTS-0.1-1.0-3H having the RC value of 72%. UV–vis analysis shows that ST(90 °C) contains ∼67% extraframework Ti species and ∼33% framework Ti species (Figure 3f). With an increase in treatment time (up to 3 h), the proportion of the framework Ti species in the TS-1 samples increased gradually. However, with a further extension of the treatment time above 3 h, the proportion of extraframework increased, and for HTS-0.1-1.0-24H anatase TiO_2_ was identified as well. N_2_ adsorption–desorption isotherms of HTS-0.1-1.0-*z*H (Figure S8) show the significant change of the hysteresis loop between HTS-0.1-1.0-2H and HTS-0.1-1.0-9H, which suggests that successful crystallization can be achieved after 9 h treatment, being in line with the XRD results. Additionally, PSDs of HTS-0.1-1.0-*z*H show that with an increase in treatment time from 3 to 9 h, with mesopore size decreases, corresponding to further crystallization and cross-growth of the nanoassembled TS-1.

**Figure 4:**
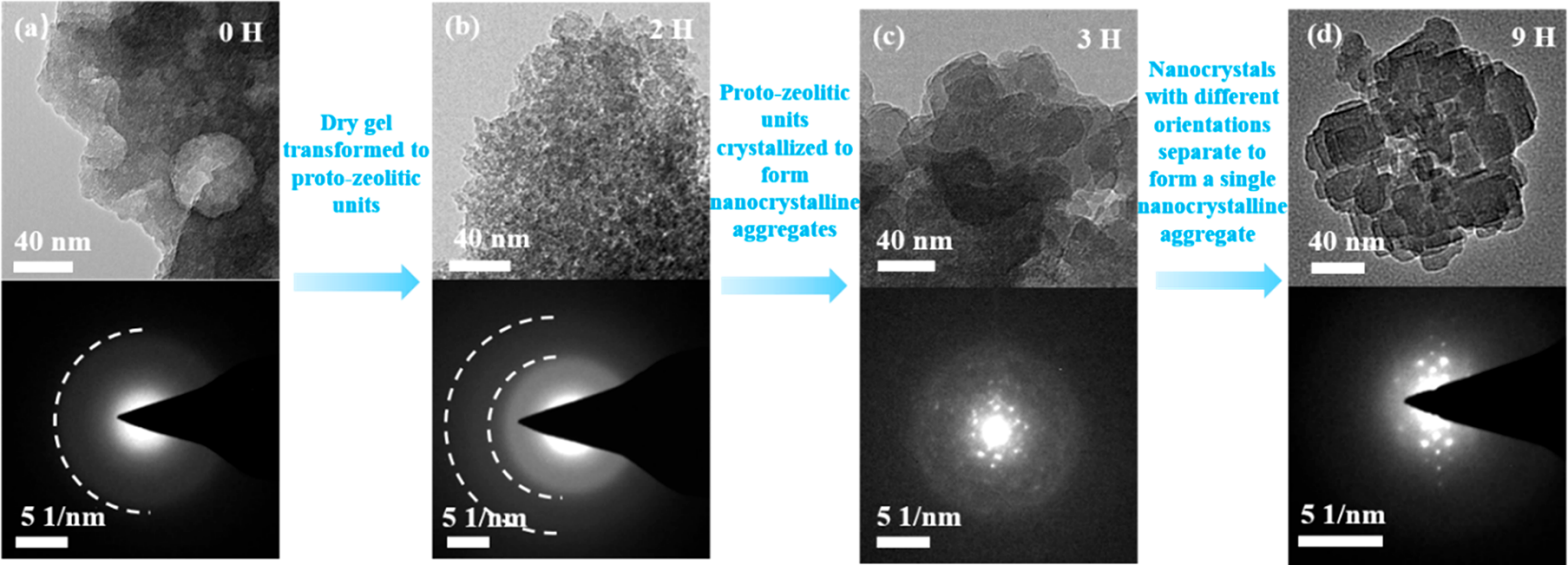
TEM micrographs and the selected area electron diffraction (SAED) of ST(90 °C) and HTS-0.1-0.1-zH.

SEM images of ST(90 °C) and HTS-0.1-0.1-*z*H are shown in Figure S9; the crushed ST(90 °C) particle has a smooth surface with some irregular voids. After the TPAOH treatment (2 h), the surface of the dry gel became rough. After 3 h, the dry gel was transformed into nanosized TS-1 crystal assemblies. Further extension of the treatment time did not bring significant changes to the morphology of the resulting materials. TEM images and the selected area electron diffraction (SAED) patterns of ST(90 °C) and HTS-0.1-0.1-*z*H (Figure 4) also revealed the crystallization process during the treatment. In detail, the diffuse diffraction rings and TEM images of ST(90 °C) show that it is amorphous with the irregular mesoporous structure (Figure 4a). After the 2-h TPAOH treatment, the SAED pattern of HTS-0.1-0.1-2H shows a broad diffraction ring, suggesting that the material has a medium-range order.^21^ In addition, the morphology of HTS-0.1-0.1-2H changed to aggregates of nanoparticles (Figure 4b). For HTS-0.1-0.1-3H, its SAED pattern shows the diffraction spots which are arranged irregularly, indicating the formation of the crystalline phase after treating the dry gel for 3 h, and the aggregates are composed of disordered accumulation of nanocrystals (Figure 4c). After a 9-h treatment, as shown in Figures 4d and S9, the diffraction spots of HTS-0.1-0.1-9H is fairly ordered, and the assembly nanocrystals exhibit lattice fringe of the same orientation, indicating that with an increase in the treatment time from 3 to 9 h, the aggregates of disordered TS-1 nanocrystals grew and assembled into densely packed zeolite crystals rather than randomly packed nanocrystal aggregates. The intracrystal mesopores in the crystals of HTS-0.1-0.1-9H can be seen in Figure S10.

**Scheme 1:**
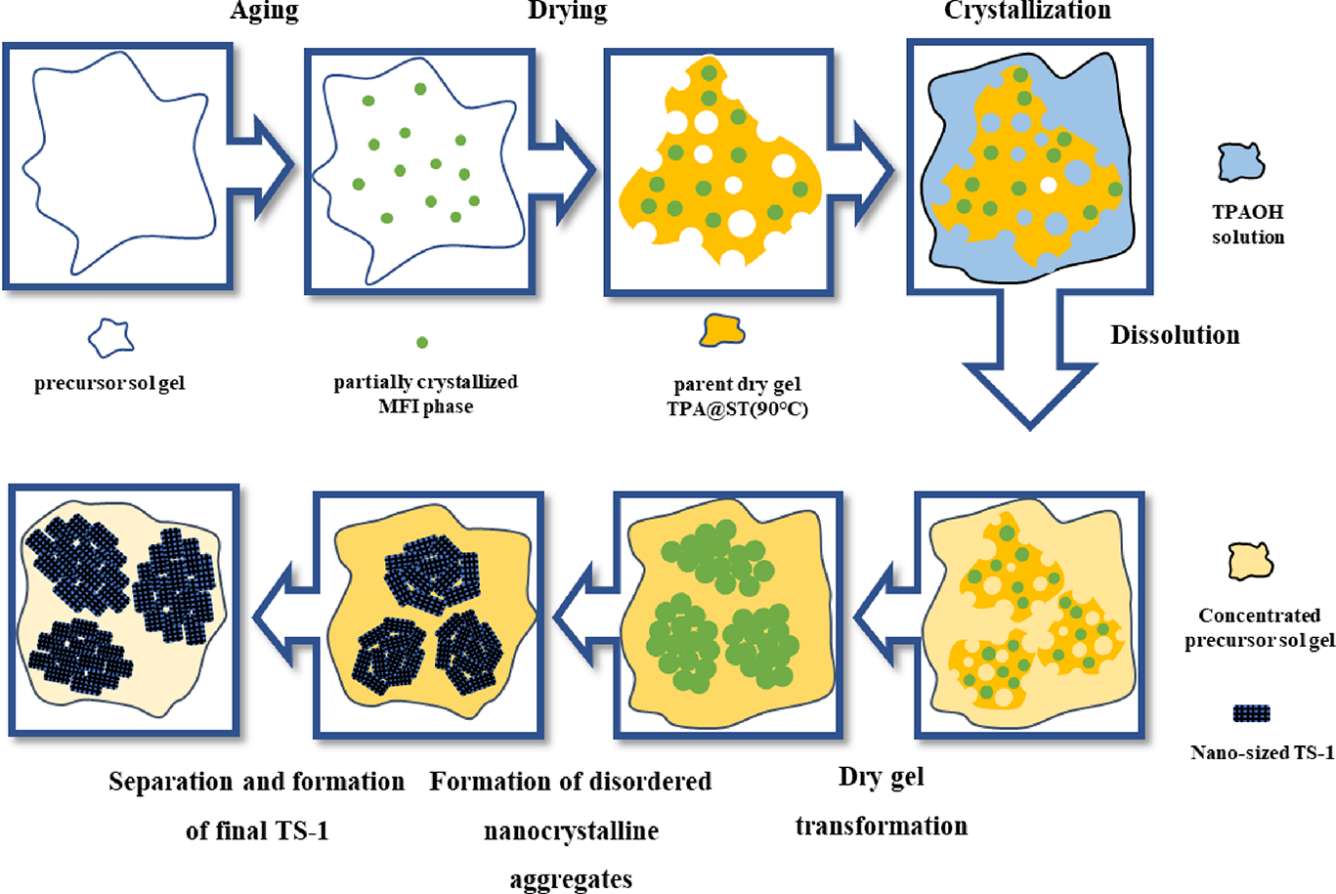
Proposed Mechanism for the Formation of Anatase-Free Nanosized Hierarchical TS-1 Using the Method Developed

Based on the results and discussion above, the mechanism of the developed method was proposed to explain the transformation of dry gel to anatase-free hierarchical TS-1, as shown in Scheme 1. In the stage of preparing the precursor, the aging process (at 90 °C for 24 h) can encourage the formation of amorphous zeolite precursor with extraframework Ti species and partial feature of MFI type zeolite. During the drying stage, the precursor shrinks to form a dry gel with many voids which can contribute to the transport of TPAOH into the dry gel in the early stage of the TPAOH treatment. During the treatment, dry gel particles are likely to be fully immersed in the TPAOH solution. The hydrothermal treatment of the preformed amorphous gel in an appropriate amount of TPAOH solution allows the fast nucleation and crystallization of TS-1 in the microzone without a substantial dissolution and reorganization of the preformed gels rich in Ti–Si bonds suppressed the formation of amorphous Ti species. Since the aged zeolite precursor dry gel has partially crystallized MFI phase, which can act as seeds, the above process can occur in a relatively short time. When the synthesis time is further increased, upon the consumption of the titanium–silicon nutrients around the newly formed nanozeolite crystals, voids (or mesopores) were formed among the nanocrystals. As the crystallization continues, the disordered nanocrystalline aggregates will adjust to be consistent as the oriented attachment growth mechanism,^37^ so that its energy can reach the minimum to form stable crystals. The above dry gel recrystallization process can also be reflected by the composition changes of the obtained samples. The Si/Ti increased first and then decreased with the extension of the crystallization time, which is due to the rapid dissolution of the unprotected extraframework Ti species in the early stage of crystallization. When the unprotected extraframework Ti species is consumed, dissolution is close to finish, and Ti species in the concentrated sol gel precursor solution are reinserted into the zeolite framework. TPAOH concentration is key to regulate the process, and the liquid-to-solid ratio is important as well to control the recrystallization which happens in the microzone during the process. Under the condition with a high TPAOH concentration, the dissolution rate of the dry gel can be much faster than the rate of crystallization, inhibiting the insertion of extraframework Ti species into the zeolite framework. When the liquid-to-solid ratio is high, the dry gel will be dissolved completely, and the zeolite crystallization process will proceed according to the liquid phase mechanism, leading to the formation of conventional large TS-1 crystals without a hierarchical structure. Since the dry gel contains incomplete crystallized zeolite, which acts as seed crystals, the recrystallization processes during the TPAOH treatment can be completed in a relatively short time. Aging of the zeolite precursor is essential for the formation of the hierarchical TS-1 zeolite since the aged zeolite precursor is known as a seed to form small zeolite crystals under conventional hydrothermal synthesis conditions.^27,38^ In addition, unlike the traditional method employing the well crystallized TS-1 for postsynthetic treatments,^14^ the developed method using the dry gel could potentially reduce the overall energy consumption for making hierarchical TS-1.

### 3.3 Catalytic Applications of the TS-1 Zeolites

Framework Ti species and their accessibility to the reactant molecules are important factors for the catalytic performance of TS-1 zeolites. In this work, hydroxylation of phenol and DBT desulfurization were employed as the model reactions to assess the catalytic ability of the TS-1 zeolites under investigation, and the obtained catalytic results are shown in Table 2. For HTS-*x*-1.7-24H, an increase in TPAOH concentration during the TPAOH treatment caused the decrease in conversions of phenol and DBT during the catalytic tests. It is well-known that in oxidation reactions using H_2_O_2_ the framework Ti species are the active sites for TS-1 catalyzed reactions, whist anatase TiO_2_ can cause decomposition of H_2_O_2_.^13,39,40^ N_2_ physisorption analysis shows that the mesopore volume and external surface area of HTS-*x*-1.7-24H TS-1 are rather comparable; hence one can infer that the decrease in the conversion of phenol is mainly due to the decrease in framework Ti species (or increase in the proportion of the sum of the extraframework Ti and anatase TiO_2_ species), which is evidenced by UV–vis analysis and ICP (Figure 3a–b, Table 1). Regarding DBT desulfurization using TBHP as the oxidizing agent over HTS-*x*-1.7-24H, the decrease in DBT conversion was rather insignificant as a function of TPAOH concentration, for example, 100% for HTS-0.1-1.7-24H vs 90.6% for HTS-0.7-1.7-24H. Previous study shows that in TS-1 the extraframework Ti species, such as hexa-coordinated Ti species, is catalytically active as well,^41^ and thus the decrease in DBT conversion is mainly due to the increase in TiO_2_ content. By comparing the catalytic performance of HTS-0.1-1.7-24H with that of HTS-0-1.7-24H, rather similar activity was measured for phenol conversion (i.e., 20.7% vs 21.0%), while the conversion of DBT over HTS-0-1.7-24H was lower than that over HTS-0.1-1.7-24H (i.e., 100.0% vs 82.3%). As shown in Figure 3, the concentrations of the framework Ti species in the two TS-1 zeolites are the same, but HTS-0.1-1.7-24H possess higher mesoporous volume and external surface area than HTS-0-1.7-24H (Table 1). Considering the kinetic sizes of phenol (kinetic diameter of 0.57 nm) and DBT (kinetic diameter of 0.9 nm), the mesoporous hierarchical HTS-0.1-1.7-24H is more beneficial to promote the catalysis involving bulk molecules such as DBT.

**Table 2:** Phenol hydroxylation and DBT Desulfurization over Various TS-1 Zeolites

		selectivity [%]			
TS-1	Con. Ph [%]^a^	CAT	HQ	PBQ	Con. DBT [%]^b^
HTS-0.1-1.7-24H	20.7	27.8	70.4	1.8	100.0
HTS-0.3-1.7-24H	19.6	31.0	66.6	2.4	97.2
HTS-0.5-1.7-24H	16.9	30.9	65.9	3.2	96.1
HTS-0.7-1.7-24H	14.4	29.2	67.2	3.6	96.0
HTS-0-1.7-24H	21.0	28.8	69.1	2.1	82.3
HTS-0.1-0.5-24H	21.0	29.1	68.5	2.4	100.00
HTS-0.1-1.0-24H	21.8	28.8	69.1	2.1	100.00
HTS-0.1-5.0-24H	19.1	25.7	71.8	2.5	97.8
ST(90 °C)	0.0	0.0	0.0	0.0	31.1
HTS-0.1-1.0-2H	0.0	0.0	0.0	0.0	100.00
HTS-0.1-1.0-3H	15.7	29.1	66.9	4.0	100.00
HTS-0.1-1.0-9H	20.8	27.7	71.3	1.0	100.00
CTS-0.1-1.0-9H	19.00	32.5	64.2	3.3	82.6
CTS-1	12.5	27.3	68.4	4.3	17.6
NTS-1	16.7	29.2	67.6	3.2	79.8

a Reaction conditions: 100 mg of catalysts; 2 g of phenol; 10 mL of water; Phenol:H_2_O_2_ = 3:1; temperature = 80 °C; reaction time = 60 min. Ph: phenol, CAT: catechol, HQ: hydroquinone, PBQ: benzoquinone.

b Reaction conditions: 50 mg of catalysts; 0.03 g of DBT; 10 mL of *n*-octane; DBT: TBHP = 1:3; temperature = 60 °C; reaction time = 90 min. DBT: dibenzothiophene.

For the catalytic performance of the TS-1 catalysts obtained from the syntheses using different liquid-to-solid ratios, that is, HTS-0.1-*y*-24H, they showed comparable activity in the two catalytic reactions, as evidenced by similar conversions of either phenol or DBT when the liquid-to-solid ratio is ≤1.7. Comparatively, HTS-0.1-5.0-24H is slightly less effective in the two reactions. The catalytic results here can be explained by the reaction characteristics of phenol hydroxylation and oxidative DBT desulfurization, as well as the pore structure and Ti coordinate states of the TS-1 catalyst. In the case in which the TS-1 zeolites contain a large amount of mesopores, the hydroxylation of phenol is mainly affected by the content of framework Ti species, and hence the reduced amount of framework Ti species in HTS-0.1-5.0-24H was responsible for the relatively low phenol conversion. For oxidative DBT desulfurization reaction, since the mesopore volume, external surface area, and framework Ti of HTS-0.1-1.7-24H and HTS-0.1-5.0-24H were lower than that of HTS-0.1-0.5-24H and HTS-0.1-1.0-24H, they showed lower DBT conversions.

Regarding the catalytic performance of the TS-1 samples (HTS-0.1-1.0-zH) obtained by varying the treatment time, the amorphous ST(90 °C) and HTS-0.1-1.0-2H are inactive in phenol hydroxylation. But when the treatment time reached 3 h, the TS-1 was found active for phenol hydroxylation, and the phenol conversion increased with an increase in the treatment time. When the treatment time exceeded 9 h, the phenol conversion over the TS-1 samples remained unchanged. Findings above are consistent with the content of the framework Ti species in HTS-0.1-1.0-*z*H (Figure S11). In DBT desulfurization, ST(90 °C) was active, and all HTS-0.1-1.0-*z*H catalysts promoted 100% DBT conversions which can be explained by the activity of the extraframework Ti species in DBT desulfurization reaction and the enhanced accessibility of the active sites due to the mesoporous dry gel.^42^

**Figure 5:**
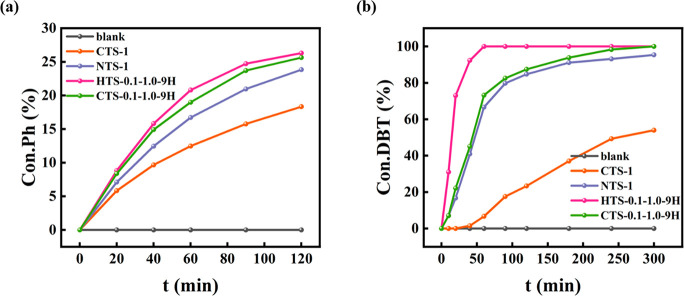
(a) Phenol hydroxylation and (b) DBT desulfurization of DBT over CTS-1, NTS-1, CTS-0.1-1.0-9H, and HTS-0.1-1.0-9H.

The effect of the aging step during the synthesis on the catalytic activity of relevant materials was also studied. As mentioned above (Section 3.1), in the conventional hydrothermal synthesis, the aged zeolite precursor promotes synthesis of TS-1 with a smaller crystal size (∼130 nm for NTS-1 vs ∼270 nm for CTS-1). The smaller zeolite crystals reduce the diffusion path and increase the accessibility of active sites, thus increasing catalytic activity (16.7% vs 12.5% for phenol hydroxylation; 79.8% vs 17.6% for DBT desulfurization). In the TPAOH treatment, the aged dry gel can be transformed into hierarchical TS-1, so HTS-0.1-1.0-9H has higher catalytic activity than CTS-0.1-1.0-9H, viz. 20.8% vs 19% for phenol hydroxylation; 100% vs 82.6% for DBT desulfurization. Moreover, in comparison with conventional hydrothermal synthesis, the TPAOH treatment enables the recrystallization of the dissolved extraframework Ti species into framework, which leads to the improved catalytic activity. The catalytic activity of the four candidates above was also studied as a function of reaction time, as shown in Figure 5. In phenol hydroxylation, after the 2-h experiments, all systems were still controlled by kinetics, and HTS-0.1-1.0-9H and CTS-0.1-1.0-9H show comparable reaction courses (Figure 5a). In DBT desulfurization, HTS-0.1-1.0-9H showed the highest activity which was reflected by the fast kinetics as a function of time on stream, i.e., 100% DBT conversion was achieved in 60 min as shown in Figure 5b. CTS-0.1-1.0-9H and NTS-1 showed a similar reaction course, and full DBT conversion was nearly achieved by the two at 300 min. Regarding CTS-1, it was the least active TS-1 catalyst in DBT desulfurization, and only about 50% DBT conversion was achieved by it after a 6-h experiment. The difference in the reaction courses in the two model reactions can be explained by the fact that DBT desulfurization is more sensitive to the presence of mesopores in TS-1 zeolites. The TON values (within the initial 20 min of the reactions) were calculated to show the intrinsic activity of the catalysts (Table S2). HTS-0.1-1.0-9H gave the highest TON for phenol hydroxylation at about 51, being higher than that of NTS-1, CTS-1, and CTS-0.1-1.0-9H (Table S2). For DBT desulfurization, CTS-1 was not active at all, whilst HTS-0.1-1.0-9H showed the highest TON of ∼6, which is four times and two times higher than that of NTS-1 and CTS-0.1-1.0-9H, respectively.

## 4 Conclusion

TS-1 zeolite is an important catalyst for many catalytic processes, and the proportion of framework Ti and anatase TiO_2_ phases of TS-1, as well as the accessibility of active sites can affect the catalytic performance of TS-1 significantly. This work presents the development of a TPAOH treatment method using the aged dry gel to prepare anatase-free hierarchical TS-1 zeolites with a high framework Ti species content. TS-1 zeolite with a Si/Ti ratio of 44, *S*_ext_ of 315 m^2^ g^–1^, and *V*_meso_ of 0.70 cm^3^ g^–1^ was successfully synthesized under the established optimum condition, viz. TPAOH concentration of 0.1 M, liquid-to-solid ratio of 1.0, and treatment time of 9 h. The development relies on the use of the aged dry gel and hydrothermal treatment using TPAOH solutions. During the synthesis of the zeolite precursor, it was found that the aging step is necessary to enable the fast nucleation and crystallization of TS-1 in the following TPAOH treatment due to the formation of partially crystallized MFI phases in the aged dry gel. During the TPAOH treatment, an appropriate TPAOH concentration and liquid-to-solid ratio could promote recrystallization of the aged dry gel to convert the extraframework Ti species in the aged dry gel into framework Ti species instead of anatase TiO_2_. In addition, the TPAOH treatment also promoted the assembly of an incomplete crystalline zeolite precursor in the aged dry gel to form nanocrystal aggregates with mesoporous structures. The TS-1 zeolites prepared by the developed method showed very good catalytic performance in phenol hydroxylation and oxidative desulfurization. In comparison with the relevant conventional methods for preparing hierarchical TS-1, the new method based on aged dry gel omitted the process to synthesize crystalline parent TS-1, hence being important for further exploration.

## Supporting Information Available

BJH pore size distributions of CTS-1, NTS-1, CTS-0.1-1.0-9H, and HTS-0.1-1.0-9H. ^29^Si NMR spectra of HTS-0.1-1.0-9H. Thermogravimetric (TG) curves of TPA@ST(90 °C). N_2_ adsorption–desorption and SEM analysis of HTS-*x*-1.7-24H and HTS-0.1-y-24H. N_2_ adsorption-desorption and SEM analysis of TPA@ST(90 °C) crystallized at different times. HRTEM analysis of HTS-0.1-0.1-9H. Calculated TON values for catalytic reactions over CTS-1, NTS-1, HTS-0.1-1.0-9H, and CTS-0.1-1.0-9H. Comparison of the physicochemical properties of relevant TS-1 zeolites (PDF)
