# Supplementary material for: Tetrapropylammonium Hydroxide Treatment of Aged Dry Gel to Make Hierarchical TS-1 Zeolites for Catalysis
Source: Cryst Growth Des. 2023 Feb 9;23(3):1775–85. doi: 10.1021/acs.cgd.2c01291 (PMC9983304; doi:10.1021/acs.cgd.2c01291)
Supplement: Supplementary file 1 — cg2c01291_si_001.pdf [file cg2c01291_si_001.pdf]

Supporting Information (SI)

TPAOH Treatment of Aged Dry Gel to Make Hierarchical TS-1 Zeolites for Catalysis

Zhenyuan Yang[1,2], Yanan Guan[1,2], Lei Xu[1], Yangtao Zhou[,1], Xiaolei Fan*[,3,4], Yilai Jiao*[,1]*

[1]Shenyang National Laboratory for Materials Science, Institute of Metal Research, Chinese Academy of Sciences, 72 Wenhua Road, Shenyang 110016, China

[2]School of Materials Science and Engineering, University of Science and Technology of China, 72 Wenhua Road, Shenyang 110016, China

[3]Department of Chemical Engineering, School of Engineering, The University of Manchester, Oxford Road, Manchester M13 9PL, United Kingdom

[4]Nottingham Ningbo China Beacons of Excellence Research and Innovation Institute, 211 Xingguang Road, Ningbo 315048, China

*Corresponding authors' emails: ytzhou@imr.ac.cn (YZ); xiaolei.fan@manchester.ac.uk (XF); yljiao@imr.ac.cn (YJ).

Supplementary Figures and Tables

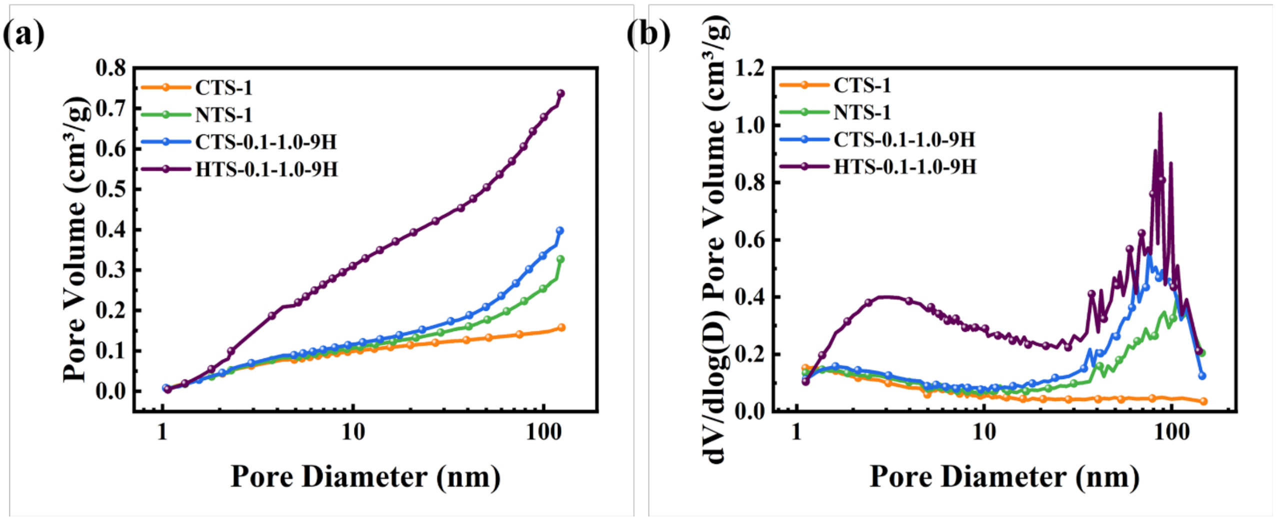

Figure S1. BJH pore size distributions of CTS-1, NTS-1, CTS-0.1-1.0-9H and HTS-0.1-1.0-9H.

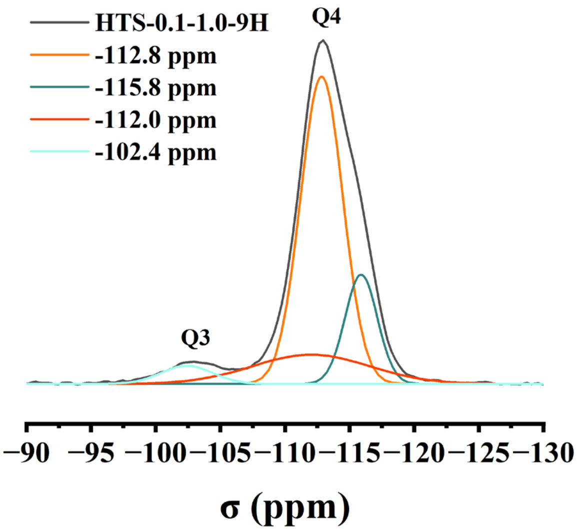

Figure S2. ^{29}Si NMR spectra of HTS-0.1-1.0-9H.

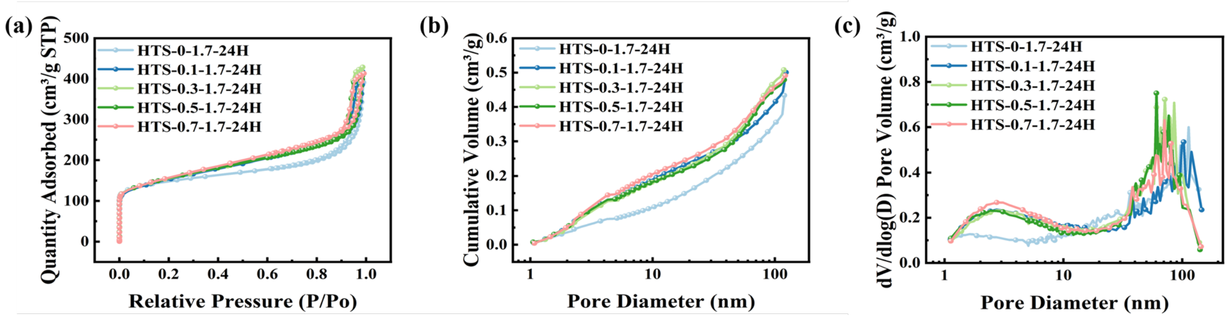

Figure S3. N_2 adsorption-desorption isotherms(a), Cumulative pore size distributions(b) and Pore size distributions(c) HTS-x-1.7-24H.

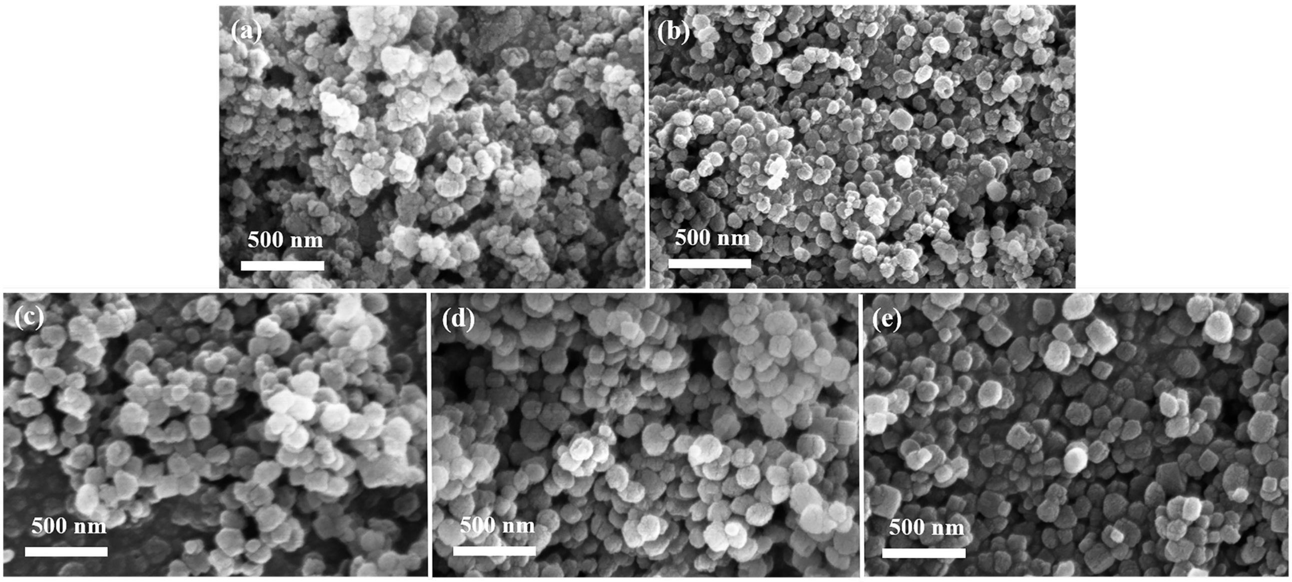

Figure S4. SEM imagines of HTS-0-1.7-24H (a), HTS-0.1-1.7-24H (b), HTS-0.3-1.7-24H (c), HTS-0.5-1.7-24H (d), HTS-0.7-1.7-24H (e).

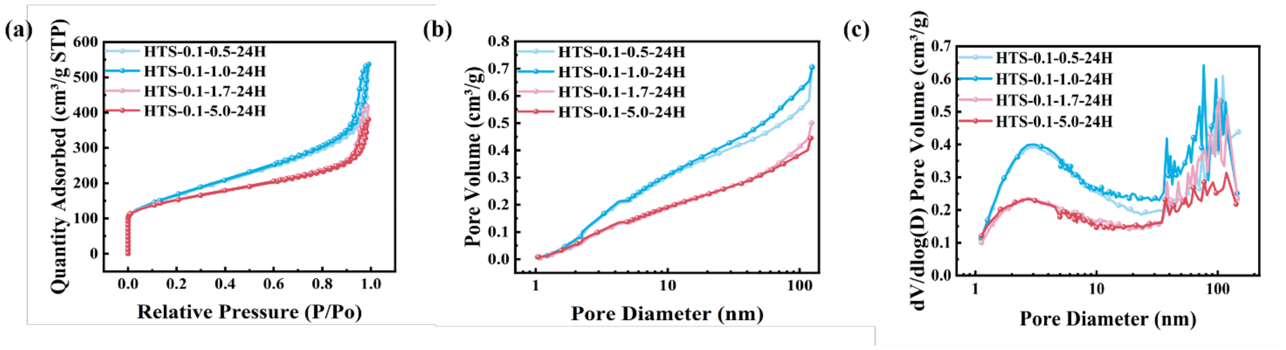

Figure S5. N_2 adsorption-desorption isotherms (a), Cumulative pore size distributions (b) and Pore size distributions (c) of HTS-0.1-y-24H.

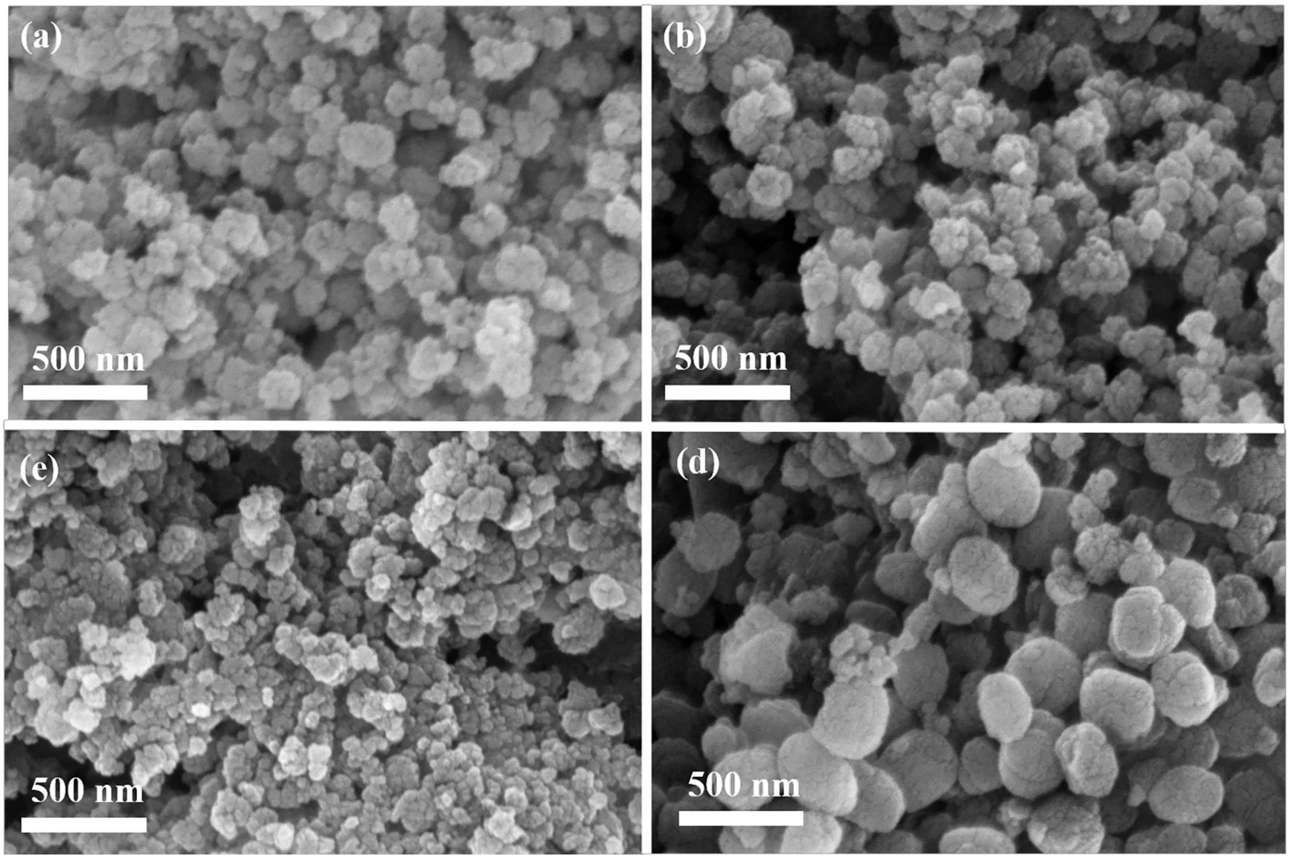

Figure S6. SEM imagines of HTS-0.1-0.5-24H (a), HTS-0.1-1.0-24H (b), HTS-0.1-1.7-24H (c), HTS-0.1-5.0-24H (d).

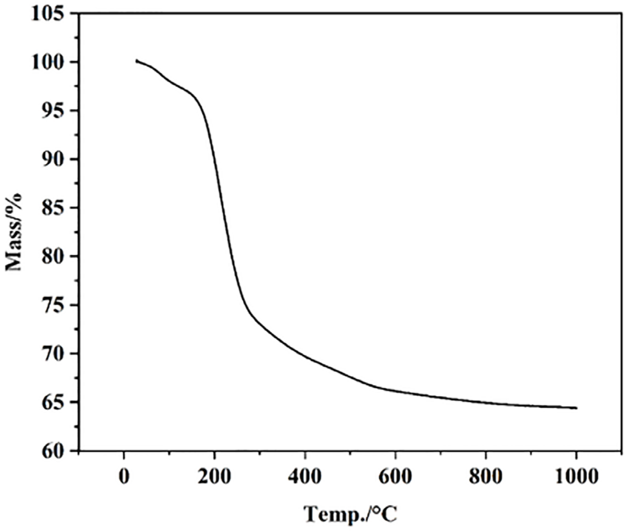

Figure S7. Thermogravimetric (TG) curves of TPA@ST(90 °C).

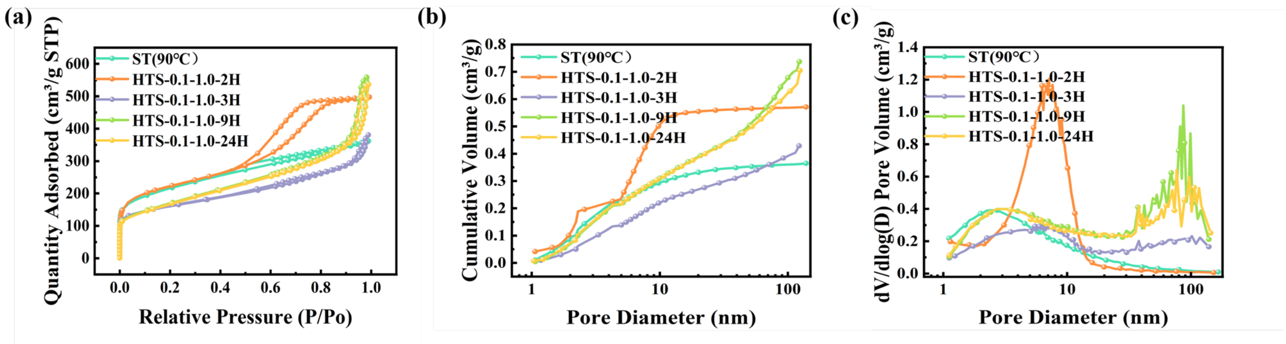

Figure S8. N_2 adsorption-desorption isotherms (a), Cumulative pore size distributions (b) and Pore size distributions (c) of TPA@ST(90 °C) crystallized at different time.

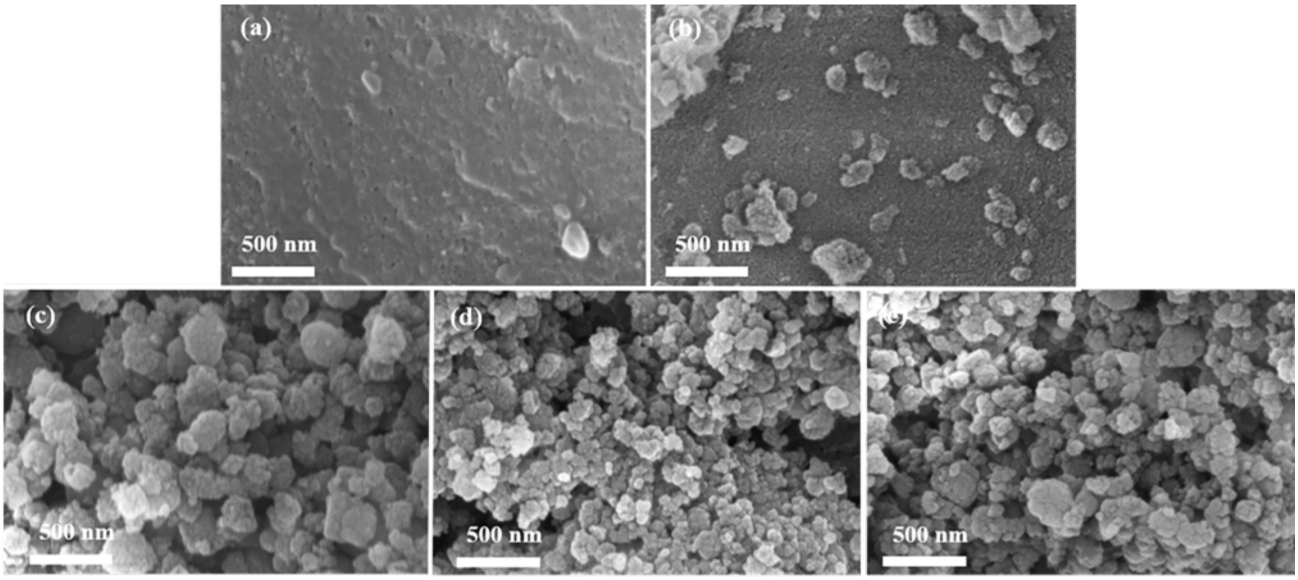

Figure S9. SEM of TPA@ST(90 °C) crystallized at different time, TPA@ST(90 °C) (a), 2 h (b), 3 h (c), 9 h (d), 24 h (e).

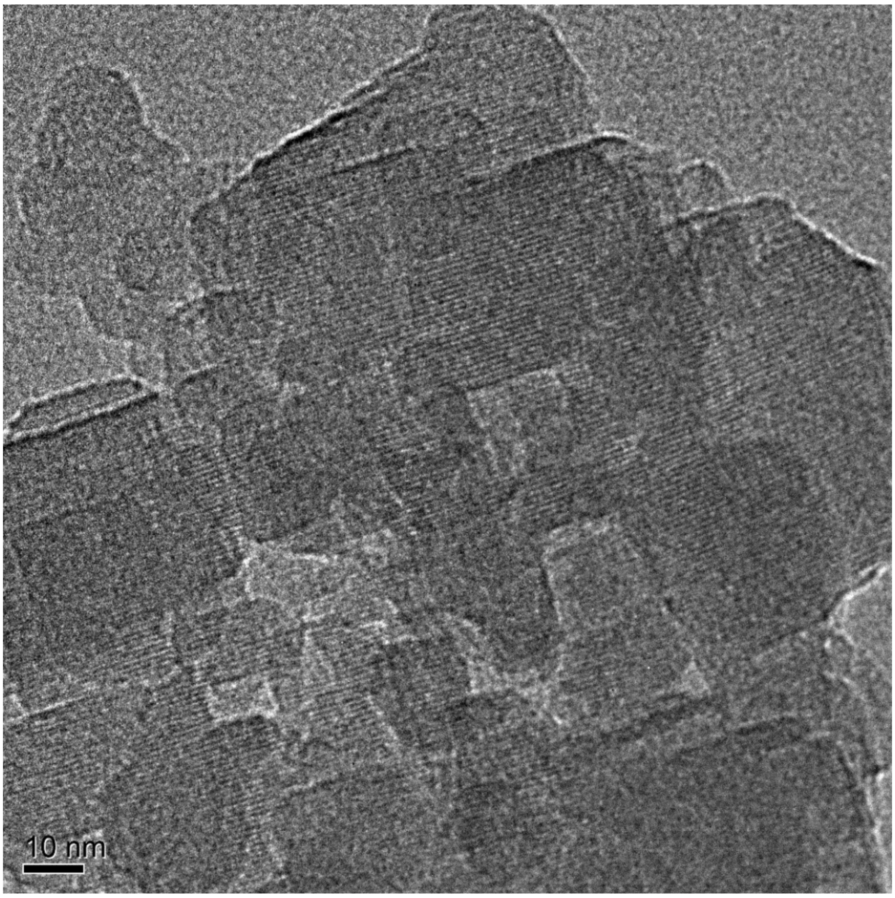

Figure S10. HRTEM images of HTS-0.1-0.1-9H.

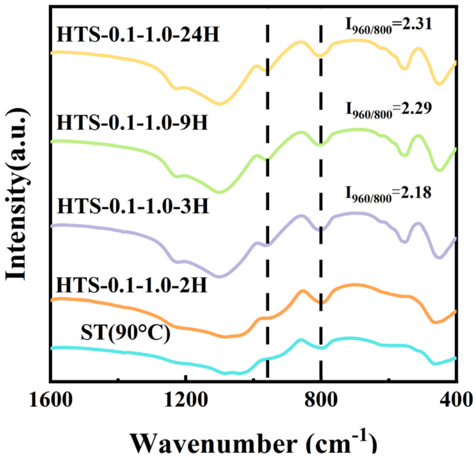

Figure S11. FT-IR spectra of HTS-0.1-0.1-zH.

Table S1. Thermogravimetric (TG) analyses of TPA@ST(90 °C).

		H_2O weight loss (%)	Template weight loss (%)	Template weight loss (%)	Total (%)
ST(90 °C)	Temperature(°C)	28-180	180-320	320-1000	35.6
	Weight loss (%)	5.96	21.79	7.85	

Table S2. TON of CTS-1, NTS-1, HTS-0.1-1.0-9H and CTS-0.1-1.0-9H.

Catalysts	CTS-1	NTS-1	HTS-0.1-1.0-9H	CTS-0.1-1.0-9H
TON.Ph	26	31	51	45
TON.DBT	0	1	6	2

Table S3. Comparison of textural properties of HTS-0.1-1.0-9H with the reference samples in the literature.

Sample	Si/Ti[a] [-]	S_{BET}[b] [$m^2 g^{-1}$]	S_{micro}[c] [$m^2 g^{-1}$]	S_{ext}[c] [$m^2 g^{-1}$]	V_{micro}[c] [$cm^3 g^{-1}$]	V_{meso}[d] [$cm^3 g^{-1}$]	Refs.
HTS-0.1-1.0-9H	44	584	269	315	0.17	0.70	This work
s-HTS-1-m(0.2,0.25)	34	426	193	233	0.20	0.38	S1
TS-1-TPAOH-6-120	35	502	373	129	0.17	0.25	S2
S-TS-1	-	378	278	100	0.12	0.10	S3
HTS-b	50	348	316	32	0.16	0.15	S4
TS-1-1-A	65	494	457	37	-	-	S5
TS-1C	39	336	129	207	0.06	0.17	S6
TS-1-T4-2	28	495	328	167	0.13	0.19	S7
HTS-1b	38	415	339	76	0.18	0.27	S8
HTS-1-60	61	473	360	113	0.19	0.28	S9
HTS-1B	22	416	277	139	0.13	0.10	S10
HTS-1C	37	413	301	122	0.14	0.17	S11

REFERENCES

S1. Han, Z.; Shen, Y.; Qin, X.; Wang, F.; Zhang, X.; Wang, G.; Li, H., Synthesis of Hierarchical Titanium-Rich Titanium Silicalite-1 Zeolites and the Highly Efficient Catalytic Performance for Hydroxylation of Phenol. ChemistrySelect 2019, 4 (5), 1618-1626.

S2. Zuo, Y.; Song, W.; Dai, C.; He, Y.; Wang, M.; Wang, X.; Guo, X., Modification of small-crystal titanium silicalite-1 with organic bases: Recrystallization and catalytic properties in the hydroxylation of phenol. Applied Catalysis A: General 2013, 453, 272-279.

S3. Tao, H.; Li, C.; Ren, J.; Wang, Y.; Lu, G., Synthesis of mesoporous zeolite single crystals with cheap porogens. Journal of Solid State Chemistry 2011, 184 (7), 1820-1827.

S4. Han, Z.; Shen, Y.; Wang, F.; Zhang, X., Synthesis of hierarchical titanium silicalite-1 in the presence of polyquaternium-7 and its application in the hydroxylation of phenol. Journal of Materials Science 2018, 53 (18), 12837-12849.

S5. Shakeri, M.; Dehghanpour, S. B., Rational synthesis of TS-1 zeolite to direct both particle size and framework Ti in favor of enhanced catalytic performance. Microporous and Mesoporous Materials 2020, 298.

S6. Du, S.; Sun, Q.; Wang, N.; Chen, X.; Jia, M.; Yu, J., Synthesis of hierarchical TS-1 zeolites with abundant and uniform intracrystalline mesopores and their highly efficient catalytic performance for oxidation desulfurization. Journal of Materials Chemistry A 2017, 5 (17), 7992-7998.

S7. Bai, R.; Song, Y.; Tian, G.; Wang, F.; Corma, A.; Yu, J., Titanium-rich TS-1 zeolite for highly efficient oxidative desulfurization. Green Energy & Environment 2021.

S8. Du, Q.; Guo, Y.; Wu, P.; Liu, H., Synthesis of hierarchically porous TS-1 zeolite with excellent deep desulfurization performance under mild conditions. Microporous and Mesoporous Materials 2018, 264, 272-280.

S9. Du, Q.; Guo, Y.; Wu, P.; Liu, H.; Chen, Y., Facile synthesis of hierarchical TS-1 zeolite without using mesopore templates and its application in deep oxidative desulfurization. Microporous and Mesoporous Materials 2019, 275, 61-68.

S10. Du, S.; Chen, X.; Sun, Q.; Wang, N.; Jia, M.; Valtchev, V.; Yu, J., A non-chemically selective top-down approach towards the preparation of hierarchical TS-1 zeolites with improved oxidative desulfurization catalytic performance. Chem Commun (Camb) 2016, 52 (17), 3580-3.

S11. Du, S.; Li, F.; Sun, Q.; Wang, N.; Jia, M.; Yu, J., A green surfactant-assisted synthesis of hierarchical TS-1 zeolites with excellent catalytic properties for oxidative desulfurization. Chem Commun (Camb) 2016, 52 (16), 3368-71.
